# Knockout of ASPP2 promotes DEN-induced hepatocarcinogenesis via the NF-κB pathway in mice

**DOI:** 10.1038/s41417-021-00300-0

**Published:** 2021-02-08

**Authors:** Shanshan Wang, Buxin Kou, Mengyin Chai, Yuxue Gao, Xuejun Lin, Ling Yin, Dexi Chen, Xiaoni Liu

**Affiliations:** Beijing Institute of Hepatology, Beijing Youan Hospital, Capital Medical University, Beijing, China

**Keywords:** Cancer genetics, Cancer models

## Abstract

Apoptosis-stimulating protein p53 2 (ASPP2) is a member of the p53-binding protein family, which is closely related to tumor development. However, the precise mechanism of ASPP2 in liver inflammation and tumorigenesis remains largely unclear. We aimed to characterize the mechanistic significance and clinical implication of ASPP2 in hepatitis and hepatocellular carcinoma (HCC). In this study, ASPP2 knockout (APKO) mice were generated to confirm the role of ASPP2 in the development of hepatitis and HCC. Liver tissues from mice were analyzed by immunohistochemistry, Western blotting, proteomic analysis, ChIP-Seq, and qRT-PCR to evaluate the role of ASPP2 in DEN-induced hepatitis and HCC. We found that APKO promoted the formation of hepatitis/hepatocarcinoma and the increased expression of proinflammatory factors. The proteomics and Western blotting results showed that APKO activated the NF-κB signaling pathway. Further, ChIP-Seq results revealed that NF-κB target genes were dramatically increased in APKO mice. In contrast, blockade of the NF-κB pathway by QNZ reduced the expression of proinflammatory factors and the susceptibility of APKO mice to DEN-induced hepatocarcinogenesis. These results suggested that the absence of ASPP2 activates the NF-κB pathway to promote the occurrence of DEN-induced hepatocarcinogenesis, indicating that ASPP2 may be a potential target for the treatment of hepatocarcinoma.

## Introduction

Hepatocellular carcinoma (HCC) is the sixth most common cancer worldwide and the third leading cause of cancer death, with 5-year overall survival rates of less than 12% [1, 2]. The pathogenesis of HCC is closely related to chronic hepatitis caused by viral infection, toxic substances, and oxidative/metabolic stress. Chronic inflammation promotes the imbalance of cell death and compensatory proliferation. The liver resists infection and toxic substance-induced hepatocellular carcinogenesis through a variety of immune regulatory mechanisms [3, 4]. Seeking therapeutic targets from the perspective of inflammation may be one of the important ways to block the occurrence of hepatocellular carcinoma, which is of great significance for the research and development of effective drugs in the treatment of hepatocellular carcinoma.

ASPP2 is one of the three members of the ASPP family. The other two members are ASPP1 and iASPP. The members of the ASPP family consist of several structural and functional domains. They are proteins containing ankyrin repeats, SH3 domains and proline-rich domain [5]. Other proteins interact with ASPP2 at different domains, and most of these interactions are mediated by the C-terminus [6]. The most well-known function of the ASPP2 protein is to regulate the apoptotic ability of p53 and its family members (p63 and p73). ASPP2 enhances the vitality of p53 family members and specifically promotes the expression of apoptotic target genes [7, 8]. In recent years, researchers have found that ASPP2 strengthens the sensitivity of drug-induced apoptosis and treatment through p53-dependent and independent pathways [9, 10]. ASPP2 also inhibits the invasion and metastasis of tumors by inhibiting epithelial interstitialization [11], epithelial polarity [12], and transforming growth factor [13, 14]. Clinical data show that the change in ASPP2 expression is related to the occurrence of many tumors, such as breast cancer, lung cancer, leukemia, gastric cancer, choriocarcinoma, and uterine cancer [15–22].

Although hepatocellular carcinoma is closely related to inflammation, it remains unclear whether ASPP2 participates in the regulation of hepatic inflammation and inhibits the occurrence of liver cancer. The NF-κB pathway is an important inflammatory signaling pathway. Computer structural simulation studies reveal that there are sites of interaction between ASPP2 and NF-κB (p65). ASPP2 (ANK-SH3) binds to p65 (amino acids 236–253 and 293–313). These sites also mediate the interaction between NF-κB and its natural inhibitory protein I-kappa B. The binding pattern of ASPP2 and NF-κB is similar to that of NF-κB and I-kappa B, which suggests that ASPP2 may be a potential inhibitor of NF-κB [23]. Another study reported that ASPP2 enhanced the p65 expression of the nuclear component by interacting with I-kappa B and mediated the inhibition of p63 expression in squamous cell carcinoma of the head and neck [24]. Therefore, we hypothesize that ASPP2 may participate in the regulation of the NF-κB pathway to inhibit inflammation, and then depress the occurrence and development of hepatocellular carcinoma. In this study, APKO mice were used to investigate the above hypothesis on the basis of a hepatitis-hepatocellular carcinoma model induced by the chemical carcinogen DEN.

## Materials and methods

### Animals and animal raising

The wild-type (WT) balb/c mice were from Beijing Vital River Laboratory Animal Technology Co., Ltd., and the APKO mice (same strain as the wild-type mice) model was generated by using the Oregon Health and Science University (OHSU) Transgenic Core [25] and conserved by the Beijing Institute of Hepatology. All mice were maintained in a sterile independent ventilation cage (IVC) system at 24–26 °C with a 12-h light/dark cycle. All animal experiments were supervised by the Laboratory Animal Management Committee of the Beijing Institute of Hepatology, and animal care was in accordance with guidelines approved by the Capital Medical University Laboratory Animal Ethics Committee.

### Animal models

Three-week-old male WT and APKO mice were intraperitoneally injected with DEN (25 mg/kg) four times once a week. Then, the mice drank 0.005% DEN ad libitum for 4 weeks to induce hepatitis and 16 weeks to establish the HCC model and began to drink normal water until 32 weeks. The 40 experimental mice were randomly divided into four groups: the WT-con group, WT-DEN group, APKO group, and APKO-DEN group.

In the second month of the establishment of the DEN-induced HCC model, QNZ (0.3 mg/kg), an inhibitor of NF-kappa B, was administered intraperitoneally once every 2 weeks for 7 consecutive months. The experimental mice were divided into two groups: WT-DEN-QNZ and APKO-DEN-QNZ.

The growth and appetite of mice were observed every day, the weight of mice was measured every week, and the death of mice was recorded.

### Magnetic resonance imaging

MRI liver images were obtained using a 3T system (MRS 3000 Benchtop MRI System). The animals were anaesthetized with 2.0% isoflurane mixed with carbogen (5% CO_2_) and maintained with 0.7–1.5% isoflurane. Axial T1-weighted (T1w) and T2-weighted (T2w) images were collected with the following parameters: FSE sequences with respiration gating; repetition time (TR)/echo time (TE)-4500/68 ms; field of view (FOV)-40.0 mm; slice thickness-1 mm, with no interspaces; acquisition time 2 min (mean), with an average of 16 slices.

### Liver histology and immunohistochemistry

Liver specimens were fixed in 10% formalin and embedded in paraffin. Four-micrometer tissue sections were stained with H&E for routine examination. First, the sections were washed in xylene and different concentrations of ethanol, distilled water, and PBS. Second, the sections were incubated in hematoxylin and eosin-G solution and rinsed with running tap water. Third, the sections were dehydrated and dewaxed. Finally, the slides were mounted with malinol. Afterwards, images were taken by microscopy.

Ki-67 was quantified using immunostaining and subsequent processing of the sections with a standard horseradish peroxidase-conjugated antibody system. In brief, tissue sections were deparaffinized and rehydrated, heat-induced epitope retrieval was performed, and endogenous peroxidase was used for blocking. Then, the cells were incubated with primary antibodies overnight at 4 °C and secondary antibodies for 60 min at room temperature. Staining was performed with 3,3′-diaminobenzidine. The immunopositive cells were counted (five fields per section, one section/mouse, and three mice/group).

### Serum analyses

Blood was preserved for further serum analyses. Blood biochemical indices, including alanine aminotransferase (ALT) and aspartate aminotransferase (AST), were measured using a Hitachi automatic analyzer. Fifty microliters of serum was used to analyze cytokines, including IL-1β, IL-6, IL-12p70, and TNFα, with a Meso Scale Discovery (MSD) kit according to the instructions.

### Proteomic analysis

Three samples from the WT-DEN and APKO-DEN groups were selected for proteomic analysis. ITRAQ (isolated tag for relative and absolute quantification) proteomic technology was used to identify differential proteins. The sample protein was extracted and evaluated by SDS-PAGE. The sample was digested with the filter-aided proteome preparation (FASP) method and labeled with a 4-plex iTRAQ Kit (AB SCIEX). The peptide segment was preseparated by high pH reversed-phase liquid chromatography, and using a C18 column. After preseparation, the partial supernatant of the sample was analyzed by liquid chromatography-mass spectrometry (Thermo™ Q Exactive HF type). The parameters were searched by Proteome Discovery.

### Western blotting

Total protein was extracted from 100 mg liver tissue samples. Protein concentrations were measured using the bicinchoninic acid method. Lysates were separated using SDS-PAGE and transferred electrophoretically to polyvinyl difluoride membranes. The membranes were blocked with tris-buffered saline containing 5% bovine serum albumin and then incubated overnight at 4 °C with primary antibodies. Then, the membranes were washed with TBST and incubated for 1 h at room temperature with the appropriate secondary antibody. The membranes were then washed, and immunoreactive bands were developed with ECL and visualized by autoradiography. Grayscale analysis of protein bands was performed using image software. Primary antibodies against ASPP2, PCNA, caspase-3, Cyclin D1, IKK, p-IKK, IκB, p-IκB, p65, p-p65, and AFP were purchased from Cell Signaling Technology.

### ChIP-Seq assay

Approximately 100 mg liver tissue was used for each ChIP-Seq assay. The chromatin precipitated by polyclonal antibodies against NF-κB were purified with the Qiagen PCR purification kit. In-depth whole-genome DNA sequencing was performed by BGI (Shenzhen, China). MACS (model-based analysis of ChIP-Seq) software was used to further analyze the ChIP-Seq data. A dynamic Poisson distribution model was used to calculate the *p*-value of the number of reads based on the unique comparison in each region to evaluate the credibility of the combined position. When the *p*-value < le−05, the region was considered a peak. Enriched binding peaks were generated after filtering through control input. For peak gene annotation, we used the KEGG database to screen and analyze all signaling pathways.

### RNA extraction and mRNA expression quantification

According to the ChIP-Seq results, the mRNA expression levels of NF-κB target genes were analyzed by real-time PCR. Total RNA was extracted, and the concentration of RNA was measured. One microgram of RNA was transcribed to cDNA using SuperScript III reverse transcriptase (Invitrogen, Life Technologies, Paisley, UK). Quantification of Frk and Nfatc1 mRNA expression was performed with real-time PCR (ABI 7300; Applied Biosystems, Foster City, CA, USA). Real-time PCR validation was carried out by using the 2-ΔΔCT method.

### Statistical analysis

Data are presented as the mean ± SD. Statistical significance of differences between groups was analyzed by unpaired Student’s *t*-test and one-way ANOVA, and *p* < 0.05 was considered to be statistically significant. Statistical significance was indicated as follows: *p < 0.05; **p < 0.01.

## Results

### DEN-treated APKO mice are prone to hepatitis formation

**Fig. 1:**
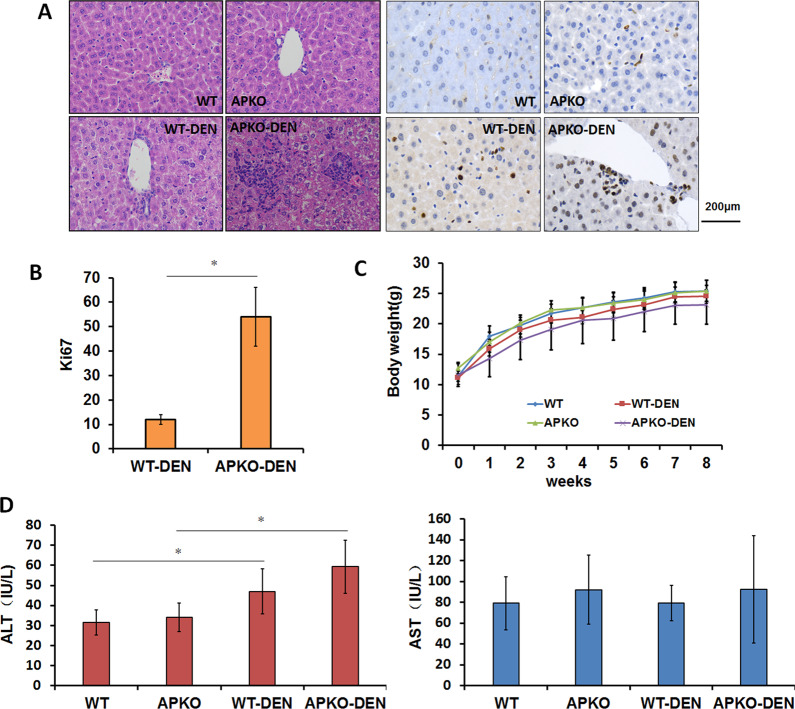
Association between APKO and inflammation in the DEN-induced hepatitis model. **A** Hematoxylin-eosin (left) and Ki67 (right) immunohistochemical staining of liver tissues. The bar graph (**B**) represents the average number of Ki-67-positive cells per field (counted in five random fields, three mice/group, one section/mouse). **C** Body weight comparison of the four groups during 8 weeks of the experiment. **D** ALT and AST were analyzed in the different groups. *p < 0.05, **p < 0.01.

The treatment of mice with DEN can simulate liver inflammation and induce liver tumorigenesis. To investigate the role of APKO in the occurrence of hepatitis, we used DEN to induce liver hepatitis in WT and APKO mice. The pathological results showed inflammatory cell infiltration and obviously increased Ki67 expression in the livers of APKO-DEN mice compared with those of WT-DEN mice (Fig. 1A, B). Compared with the WT-DEN group, the APKO-DEN group had markedly decreased body weight (Fig. 1C). To evaluate liver function and integrity, liver injury parameters, such as ALT and AST, were measured (Fig. 1D). Plasma ALT was elevated by DEN treatment, but the increase in the APKO-DEN group was greater than that in the WT-DEN group. These data showed that APKO mice induced by DEN are more prone to liver damage, which can lead to severe hepatitis and even to HCC.

### APKO mice induced by DEN are more likely to develop hepatocarcinogenesis than WT mice

Inflammation plays a key role in the development of liver cancer. To further investigate the role of APKO in DEN-induced liver damage, we used DEN to induce a liver tumor model in WT and APKO mice.

**Fig. 2:**
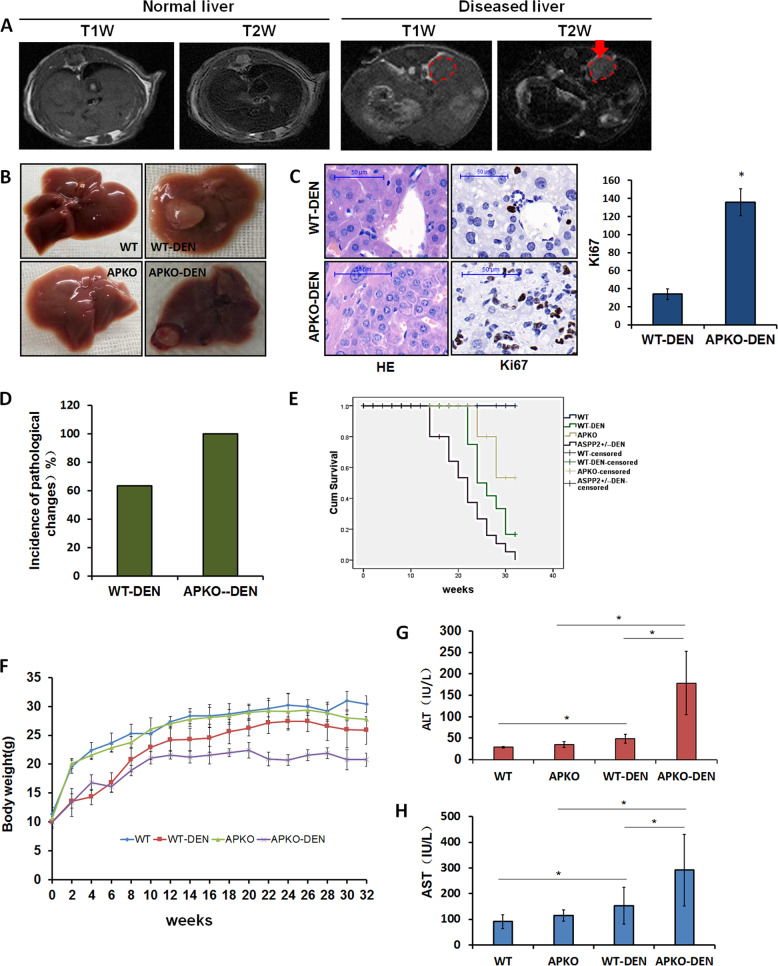
The susceptibility of APKO mice to DEN-induced hepatocarcinogenesis was dramatically increased compared to that of wild-type mice. **A** MRI images: axial view of MRI T1-weighted and T2-weighted images of normal and DEN-treated livers. The red circle denotes tumors. **B** Gross liver morphology of mice. The arrows point to tumors. **C** Hematoxylin-eosin and Ki67 immunohistochemical staining of liver tissues. The bar graph represents the average number of Ki-67-positive cells per field (counted in five random fields, three mice/group, one section/mouse). **D** Incidence of liver tumorigenesis in mice in the APKO-DEN and WT-DEN groups. **E** Survival rates of mice from different groups. **F** Body weight comparison of the four different groups during the 32-week experiment. **G**, **H** Plasma parameters indicating liver injury, such as ALT and AST, were analyzed in the different groups. *p < 0.05, **p < 0.01.

First, we used a magnetic resonance instrument to monitor tumor growth 32 weeks after DEN injection before sacrificing the animals. The liver lesions were characterized by high heterogeneity in signal intensity on T1-weighted images, contrasting the homogeneous normal liver tissue. T2-weighted images showed that the tumors had a high intensity, while the normal liver tissue had a low intensity (Fig. 2A). Gross observation indicated that the mice treated with DEN for 32 weeks developed liver tumors. Neither APKO mice nor WT control mice developed spontaneous tumors when injected with saline (Fig. 2B). Ki67-positive cells were greatly increased in the HCC tissues in APKO-DEN mice compared to WT-DEN mice (Fig. 2C). The incidence of tumorigenesis in the APKO-DEN group (100%) was higher than that in the WT-DEN group (63.3%) (Fig. 2D). The survival rates of mice in the WT, WT-DEN, APKO, and APKO-DEN groups were 100.0%, 56.5%, 88.2%, and 29.6%, respectively (Fig. 2E). The results showed that the body weight change in the APKO-DEN group was significantly greater than that in the other groups (Fig. 2F). To evaluate liver function and liver injury, ALT, and AST were measured (Fig. 2G, H). Plasma ALT and AST were increased in the APKO-DEN group compared with the WT-DEN group. These data confirmed that the APKO mice induced by DEN are markedly more likely to develop hepatocarcinogenesis than WT mice.

### APKO activates the NF-κB signaling pathway in DEN-induced mice with hepatocarcinoma

**Fig. 3:**
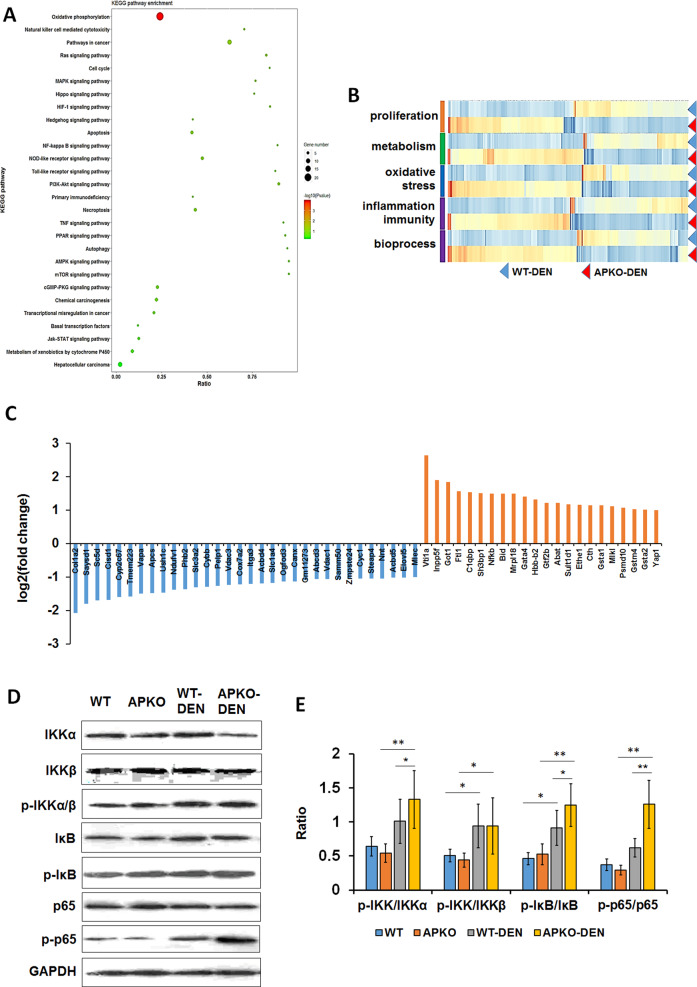
Proteomic analysis of livers from DEN-induced APKO and WT hepatocarcinoma mice. **A** KEGG pathway annotation of differentially expressed proteins indicated that ASPP2 is involved in the NF-κB pathway. **B** A total of 4609 proteins were changed in the APKO versus WT mouse group comparison, and most of the changed proteins were involved in cell proliferation, metabolism, oxidative stress, inflammation and biological processes. **C** According to the standard of fold change > 2 and *p* < 0.05, 32 downregulated and 23 upregulated proteins are listed. **D** Western blotting (**D**) and statistical (**E**) analysis of the expression of NF-κB pathway-related proteins. *p < 0.05, **p < 0.01.

To explore the precise molecular mechanism of ASPP2 in DEN-induced hepatocarcinogenesis, we used proteomics to identify the differentially expressed proteins in the liver cancer tissues in the WT-DEN and APKO-DEN groups. A total of 4609 proteins were identified, mainly involving proliferation, metabolism, oxidative stress, inflammation, and biological processes (Fig. 3B). Based on the analysis, we identified 32 downregulated and 23 upregulated proteins (Fig. 3C). All the changed proteins were analyzed by KEGG pathway analysis, and the results showed that more than 20 pathways were changed in the APKO-DEN-induced HCC group versus WT-DEN group comparison (Fig. 3A). Among these pathways, we identified cancer-related signaling pathways, including the NF-kappa B (NF-κB) signaling pathway, peroxisome proliferator-activated receptor (PPAR) signaling pathway, PI3K-Akt signaling pathway, and TNF signaling pathway.

According to a previous description, ASPP2 is closely related to NF-κB, so we chose the NF-κB pathway for further verification. Western blotting results showed that p-IKK, p-IκB, and p-p65 expression was significantly upregulated and that the p-IKK/IKKα, p-IκB/IκB, and p-p65/p65 ratios were obviously increased in the APKO-DEN group compared with the WT-DEN group (Fig. 3D, E), which suggested that APKO activated the NF-κB pathway.

### The expression of proinflammatory factors and tumor-related proteins was upregulated in APKO DEN-induced HCC mice

Inflammatory factors play an important role in the occurrence and development of HCC. Therefore, we analyzed several important proinflammatory cytokines in these four groups of mice.

**Fig. 4:**
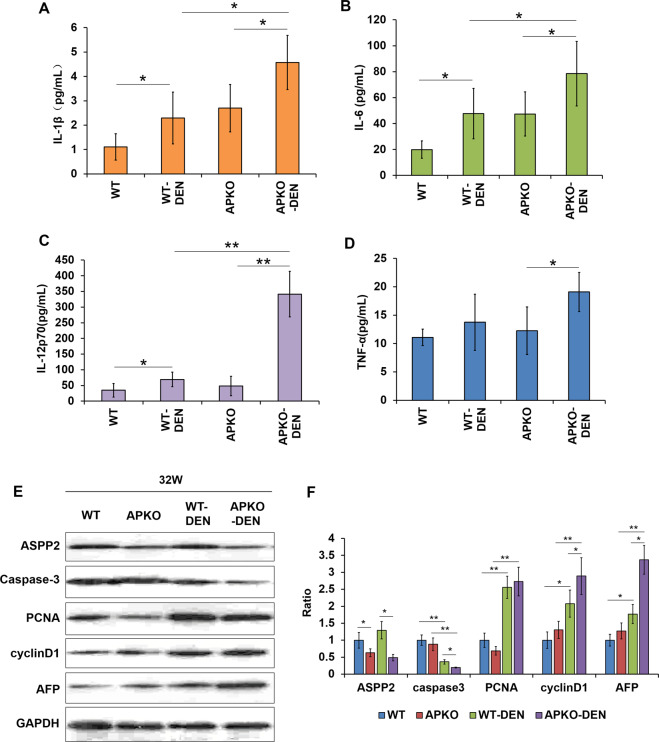
Proinflammatory factor and tumor-related protein expression levels were changed in APKO mice. IL-1β (**A**), IL-6 (**B**), IL-12p70 (**C**) and TNFα (**D**) expression levels were measured in the different groups of mice. **E** Western blotting (E) and statistical analysis (**F**) of the expression of ASPP2, caspase-3, PCNA, cyclinD1 and AFP in the liver tissues. *p < 0.05, **p < 0.01.

We found a significant increase in inflammatory factors in the blood of WT and APKO DEN-induced HCC mice. Among them, the levels of IL-1β (Fig. 4A), IL-6 (Fig. 4B), and IL-12p70 (Fig. 4C) in the blood of the APKO-DEN group were significantly higher than those in the blood of the WT-DEN group, but no obvious difference was observed in TNFα (Fig. 4D) between the two groups. All these results illuminated that the loss of ASPP2 promoted the development of liver inflammation.

Additionally, we evaluated the expression levels of some tumor-related proteins by Western blotting in the three liver tissues of each group. The expression of caspase-3 was significantly decreased, and PCNA, cyclinD1, and AFP were increased in the APKO-DEN group compared with the WT-DEN group (Fig. 4E, F). This result suggested that APKO promoted DEN-induced inflammation in the liver.

### The absence of ASPP2 resulted in changes in the regulation of NF-κB target genes in APKO DEN-induced HCC mice

To identify whether the absence of ASPP2 influences NF-κB-regulated target genes, we conducted genome-wide mapping of NF-κB binding sites in the WT and APKO DEN-induced HCC groups by ChIP-Seq.

**Fig. 5:**
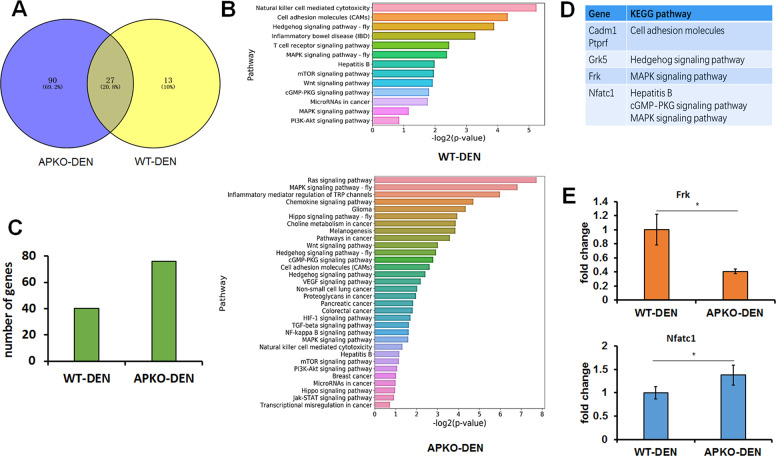
ChIP-Seq analysis of NF-κB target genes. To identify direct NF-κB target genes, we conducted genome-wide mapping of NF-κB binding sites in the APKO-DEN and WT-DEN groups. **A** A Venn diagram showing the number of pathways involving NF-κB target genes in the APKO-DEN and WT-DEN groups. **B** List of the pathways related to the development of tumors in the WT-DEN (up) and APKO-DEN (down) groups. **C** The diagram shows the number of enriched genes in the two groups according to the pathway results. **D** List of five selected NF-κB target genes and symbols. **E** RT-qPCR analysis of the expression of the Frk and Nfatc1 genes in the WT-DEN and APKO-DEN groups. *p < 0.05.

A Venn diagram showed that in the APKO-DEN group, NF-Κb target genes were enriched in 117 signaling pathways compared with the 40 enriched signaling pathways in the WT-DEN group (Fig. 5A). Further analysis showed that in the WT-DEN group, there were 13 signaling pathways related to tumors, and in the APKO-DEN group, there were 32 signaling pathways related to tumors (Fig. 5B). Among these tumor-related pathways, 40 genes were found in the WT-DEN group, and 75 genes were found in the APKO-DEN group (Fig. 5C). Five target genes (Cadm1, Ptprf, Grk5, Frk, and Nfatc1) were selected and listed from 11 intersecting signaling pathways related to tumors for further qRT-PCR verification (Fig. 5D). The results revealed that the expression of the Frk gene (tumor suppressor gene) was significantly decreased, and the expression of the Nfatc1 gene (tumor promoter gene) was significantly increased in the APKO-DEN group compared with the WT-DEN group (Fig. 5E). These results suggested that APKO affected the transcription of NF-κB target genes, especially genes related to tumors.

### The proinflammatory factors and NF-κB signaling pathway were evaluated in APKO-DEN-induced hepatitis mice

Previous results showed that APKO upregulated proinflammatory factors and activated the NF-κB signaling pathway in HCC mice. To further investigate the role of ASPP2 in hepatitis, we analyzed the expression of proinflammatory factors and NF-κB signaling pathway-related proteins in APKO DEN-induced hepatitis mice.

**Fig. 6:**
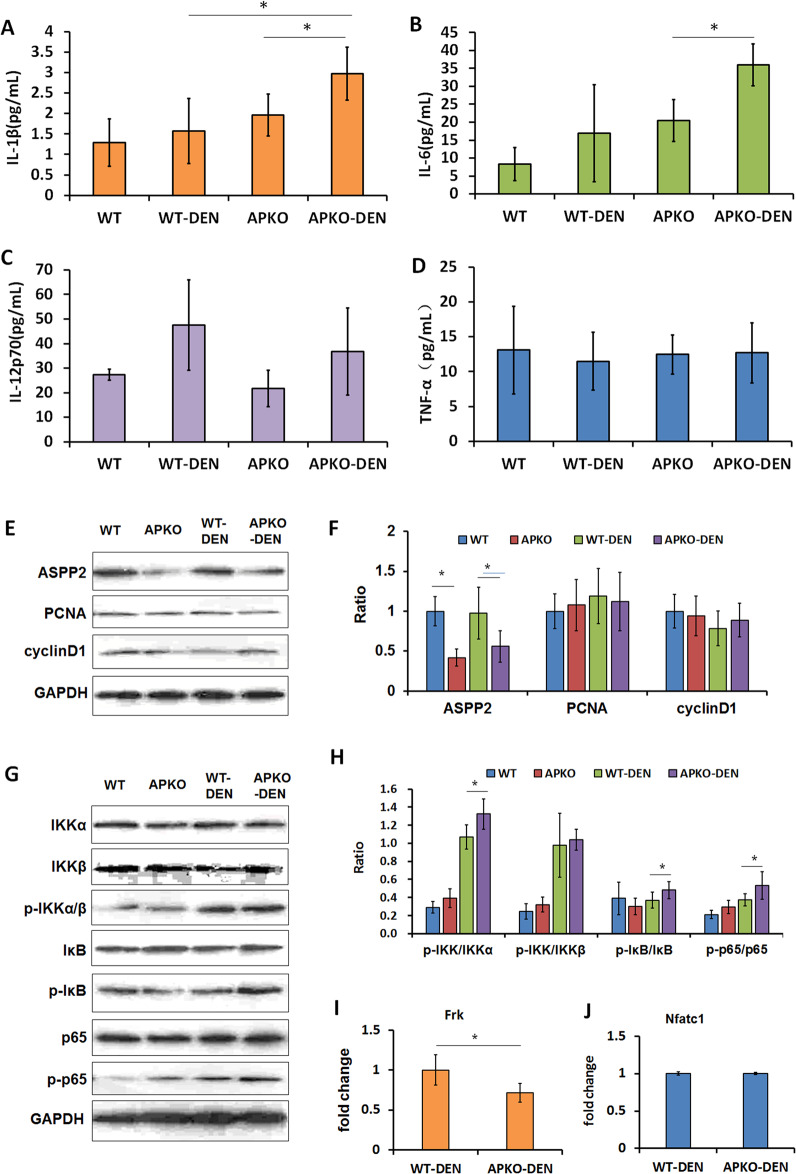
Effects of APKO in the DEN-induced hepatitis model. IL-1β (**A**), IL-6 (**B**), IL-12p70 (**C**), and TNFα (**D**) expression was measured in the different groups of mice. Western blotting (**E**) and statistical analysis (**F**) of the expression of ASPP2, PCNA, and cyclinD1 in liver tissues. Western blotting (**G**) and statistical analysis (**H**) of the expression of NF-κB pathway-related proteins. RT-qPCR analysis of the expression of the Frk (**I**) and Nfatc1 (**J**) genes in the WT-DEN and APKO-DEN groups. *p < 0.05.

The results showed that IL-1β and IL-6 levels were increased in the APKO-DEN hepatitis mice compared with WT-DEN mice (Fig. 6A, B), but the IL-12p70 and TNFα levels did not change significantly (Fig. 6C, D). In addition to these proinflammatory factors, we also analyzed the expression of tumor proliferation-related proteins (PCNA and cyclin D1). The Western blotting results showed that there was no significant difference among the different groups (Fig. 6E, F). However, the NF-κB signaling pathway was still activated. Compared with the WT-DEN group, the APKO-DEN group showed significantly upregulated p-IKK, p-IκB, and p-p65 expression and obviously increased p-IKK/IKKα, p-IκB/IκB and p-p65/p65 ratios (Fig. 6G, H). The Frk and Ncfc1 genes were also analyzed in the WT-DEN and APKO-DEN hepatitis groups. qRT-PCR results showed that the Frk gene was significantly decreased in the APKO-DEN group (Fig. 6I), but the Ncfc1 gene was not changed (Fig. 6J). These results suggested that in the DEN-induced hepatitis mouse model, the absence of ASPP2 promoted the development of liver inflammation and activated the NF-κB signaling pathway.

### APKO attenuates DEN-induced hepatocarcinogenesis by blocking the NF-κB pathway

To further verify that APKO promotes DEN-induced hepatocarcinogenesis by activating the NF-κB pathway, QNZ (an inhibitor of the NF-κB pathway) was injected intraperitoneally in the process of DEN-induced liver cancer formation in mice.

**Fig. 7:**
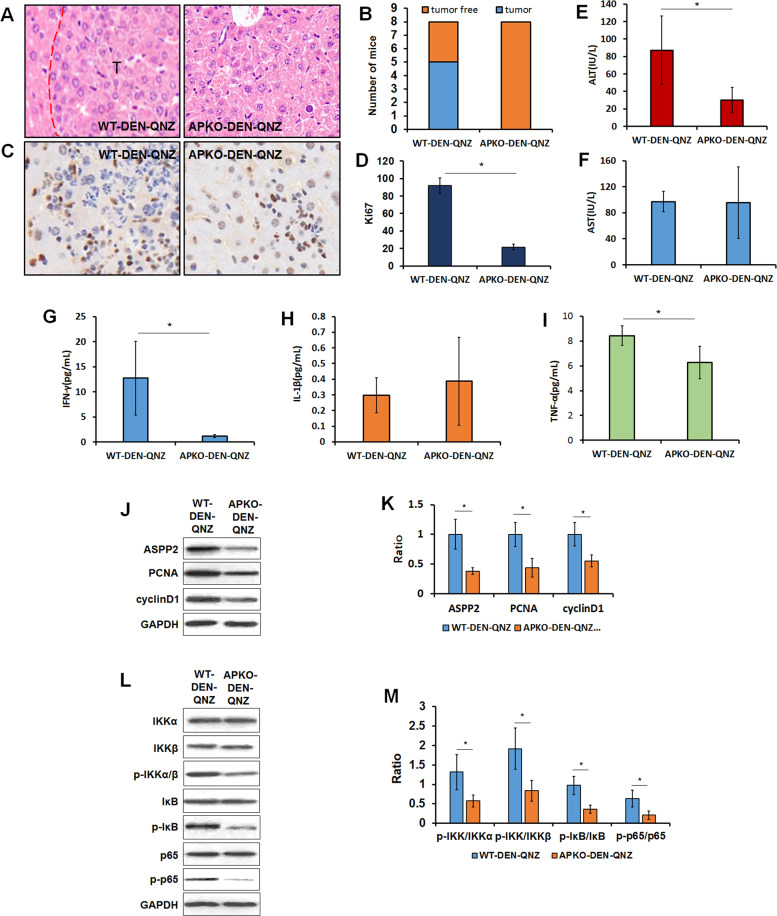
Inhibition of the NF-κB pathway reduced the susceptibility of APKO mice to DEN-induced hepatocarcinogenesis. Hematoxylin-eosin (**A**) and Ki67 (**C**) immunohistochemical staining of liver tissues in the WT-DNE-QNZ and APKO-DEN-QNZ groups. The bar diagram (**D**) represents the average number of Ki-67-positive cells per field. **B** The diagram shows the number of tumor-containing and tumor-free mice in these two groups. ALT (**E**) and AST (**F**) were measured in the different groups. IL-1β (**G**), IL-6 (**H**), and TNFα (**I**) expression was measured in the WT-DNE-QNZ and APKO-DEN-QNZ groups. Western blotting (**J**) and statistical analysis (**K**) of the expression of ASPP2, PCNA, and cyclinD1 in liver tissues. Western blotting (**L**) and statistical analysis (**M**) of the expression of NF-κB pathway-related proteins. *p < 0.05.

HE results showed that compared with the WT-DEN-QNZ group, the APKO-DEN-QNZ group had a decreased incidence of tumor formation (Fig. 7A, B). The number of Ki67-positive cells was decreased greatly in the HCC tissues of APKO-DEN- QNZ mice compared with those of WT-DEN QNZ mice (Fig. 7C, D). Liver function was analyzed in these two groups. The results showed that the ALT level was extremely decreased in the APKO-DEN-QNZ group, and the AST level was not changed (Fig. 7E, F). The expression levels of the inflammatory factors IFN-ɤ (Fig. 7G), IL-1β (Fig. 7H), and TNFα (Fig. 7I) were also reduced in the APKO-DEN-QNZ group. These results suggest that QNZ injection can reduce the incidence and malignancy of DEN-induced HCC in APKO mice. Similar results were observed for tumor proliferation-related proteins (PCNA and cyclin D1) and NF-κB pathway-related proteins (p-IKK, p-IκB, and p-p65), which were decreased in the APKO-DEN-QNZ group. Together, these results suggest that APKO indeed activates the NF-κB pathway, which promotes the inflammation of liver tissue and leads to the occurrence of hepatocarcinoma.

## Discussion

ASPP2 has a wide range of biological functions, including apoptosis, autophagy, and the inflammatory response [26]. Recent studies have shown that ASPP2 is a potential tumor suppressor that can enhance sensitivity to liver cancer drugs, but its role in hepatocarcinogenesis is not clear [27]. The occurrence of liver cancer is closely related to inflammation. ASPP2 may play a dual role in liver inflammation. A report showed that haploid deletion of ASPP2 protected against acute liver injury induced by CCL4 by activating autophagy and inhibiting inflammation and apoptosis [28]. In a model of LPS-induced inflammation in female rats, LPS induced the expression of ASPP2 by passing through the blood brain spinal fluid barrier (brain inflammatory barrier), which mediates LPS-induced apoptosis. Consistent with the role of ASPP2 as an inflammatory gatekeeper, the ASPP2-deficient brain shows enhanced neuroinflammation [29].

What role does ASPP2 play in hepatocarcinogenesis? Is ASPP2 related to inflammation? In vitro study, our research team have reported that overexpress ASPP2 in the HCC cell lines, which induced cells apoptosis in the previous researches [30, 31]. To further study the role and mechanism of ASPP2 in hepatocarcinogenesis in vivo, we established a DEN-induced hepatocarcinogenesis model based on APKO mice. The results revealed that the rates of hepatocarcinogenesis in APKO mice were significantly higher than those in wild-type mice, obvious liver function damage occurred, and the levels of proinflammatory factors and proliferative tumor proteins in the liver were significantly increased when DEN was continuously administered for 32 weeks. The NF-κB pathway is one of the important inflammatory signaling pathways and is also a key mediator between inflammation and cancer [32]. NF-κB is activated in the liver cancer tissue of patients, which is closely related to the occurrence, progression, and prognosis of liver cancer [33, 34]. Our proteomics and WB results showed that the NF-κB pathway was activated in APKO mice after DEN treatment. ChIP-Seq data showed that the expression of NF-κB target genes in APKO mice was significantly different from that in wild-type mice. Frk (Fyn-related kinase) is a member of a small family of Src-related tyrosine kinases that includes PTK6 and Srms. Frk mediates the tyrosine phosphorylation of PTEN, inhibiting the association of PTEN with NEDD4-1 and further suppressing tumors [35, 36]. NFATc1 (nuclear factor of activated T cells) is a member of the NFAT family and a key regulator of the immune response. NFATc1 overexpression can promote the occurrence, progression, and metastasis of a variety of tumors and is a poor prognostic factor [37, 38]. Both Frk and NFATc1 are downstream target genes of NF-κB. In this study, the Frk gene was significantly downregulated and the NFATc1 gene was significantly upregulated after 32 W of DEN treatment in APKO mice. These results suggested that the loss of ASPP2 promoted DEN-induced hepatocarcinogenesis in mice, which might be caused by the activation of the NF-κB pathway and by the regulation of the expression of inflammatory factors and tumor-related genes in the livers.

We also observed the livers of APKO mice treated with DEN for 8 weeks. We found that inflammatory cell infiltration and inflammatory factor and Ki67 expression were increased, while NF-κB pathway activation and Frk gene expression were decreased in the APKO-DEN group compared with the WT-DEN group. These results suggested that APKO promoted DEN-induced inflammation in the mouse liver, which may be the basis for the further development of liver cancer.

To further verify whether APKO activated the NF-κB pathway, QNZ (an inhibitor of the NFkB pathway) was administered to APKO mice and wild-type mice at the same time during DEN treatment. QNZ was found to significantly reduce the formation of liver cancer in APKO mice. It was further confirmed that ASPP2 deficiency markedly activated the NF-κB pathway, regulated the expression of inflammatory factors and tumor-related genes in the liver, and promoted DEN-induced hepatocarcinogenesis in mice.

**Fig. 8:**
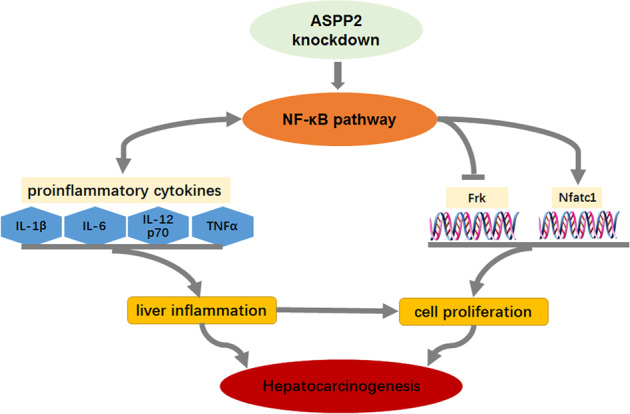
Schematic model of the possible role of ASPP2 in hepatitis and hepatocarcinogenesis. Sumrized the role of ASPP2 via NF-κB pathway to participate the hepatocarcinogenesis. →: Postive regulation; ↔: Bi-directional regulation; —Ι: Negative regulation.

In conclusion, our findings reveal that ASPP2 deficiency is a key driver of DEN-induced hepatocarcinogenesis. In this disorder, the loss of ASPP2 can affect the expression of inflammatory factors and tumor-related genes by activating the NF-κB pathway and can promote inflammation in the liver to support the occurrence of cancer, as shown in Fig. 8.
